# The use of yellow fluorescent hybrids to indicate mating in *Trypanosoma brucei*

**DOI:** 10.1186/1756-3305-1-4

**Published:** 2008-02-25

**Authors:** Wendy Gibson, Lori Peacock, Vanessa Ferris, Katherine Williams, Mick Bailey

**Affiliations:** 1 School of Biological Sciences University of Bristol, Bristol BS8 1UG, UK; 2 Department of Clinical Veterinary Science, University of Bristol, Langford, Bristol BS40 7DU, UK

## Abstract

**Background:**

*Trypanosoma brucei *undergoes genetic exchange in its insect vector, the tsetse fly, by an unknown mechanism. The difficulties of working with this experimental system of genetic exchange have hampered investigation, particularly because the trypanosome life cycle stages involved cannot be cultured in vitro and therefore must be examined in the insect. Searching for small numbers of hybrid trypanosomes directly in the fly has become possible through the incorporation of fluorescent reporter genes, and we have previously carried out a successful cross using a reporter-repressor strategy. However, we could not be certain that all fluorescent trypanosomes observed in that cross were hybrids, due to mutations of the repressor leading to spontaneous fluorescence, and we have therefore developed an alternative strategy.

**Results:**

To visualize the production of hybrids in the fly, parental trypanosome clones were transfected with a gene encoding Green Fluorescent Protein (GFP) or Red Fluorescent Protein (RFP). Co-infection of flies with red and green fluorescent parental trypanosomes produced yellow fluorescent hybrids, which were easily visualized in the fly salivary glands. Yellow trypanosomes were not seen in midgut or proventricular samples and first appeared in the glands as epimastigotes as early as 13 days after fly infection. Cloned progeny originating from individual salivary glands had yellow, red, green or no fluorescence and were confirmed as hybrids by microsatellite, molecular karyotype and kinetoplast (mitochondrial) DNA analyses. Hybrid clones showed biparental inheritance of both nuclear and kinetoplast genomes. While segregation and reassortment of the reporter genes and microsatellite alleles were consistent with Mendelian inheritance, flow cytometry measurement of DNA content revealed both diploid and polyploid trypanosomes among the hybrid progeny clones.

**Conclusion:**

The strategy of using production of yellow hybrids to indicate mating in trypanosomes provides a robust and unequivocal system for analysis of genetic exchange. Mating occurred with high frequency in these experimental crosses, limited only by the ability of both parental trypanosomes to invade the salivary glands. Yellow hybrids appeared as soon as trypanosomes invaded the salivary glands, implicating the short, unattached epimastigote as the sexual stage. The recovery of diploid, triploid and tetraploid hybrids in these crosses was surprising as genetic markers appeared to have been inherited according to Mendelian rules. As the polyploid hybrids could have been produced from fusion of unreduced gametes, there is no fundamental conflict with a model of genetic exchange involving meiosis.

## Background

Trypanosomes (Euglenozoa: Kinetoplastea [1]) are widespread and ubiquitous parasites of vertebrates, but the best known species are those that cause disease in humans and domestic livestock. So far genetic exchange has been demonstrated experimentally in two species, *Trypanosoma brucei *[2] and *T. cruzi *[3]. However, details of the mechanism remain elusive and the frequency of genetic exchange in nature is controversial [4,5]. Determining how trypanosomes achieve genetic exchange is not only important for understanding gene flow in these pathogens, but also has relevance to the study of the early evolution of eukaryotes, as trypanosomes arise from a deep branch of the eukaryote tree [6].

Genetic exchange is not an obligatory part of the trypanosome life cycle and, for example, occurs only in a proportion of experimental flies co-infected with two different *T. brucei *strains [2,7]. *T. brucei *undergoes a complex life cycle involving both mammalian and bloodsucking insect (tsetse fly) hosts [8]. Bloodstream form trypanosomes, taken up by the fly as it feeds, first differentiate into procyclic forms and multiply within the midgut, before moving forward to invade the salivary glands via the foregut and mouthparts [9]. Genetic exchange most likely occurs in the fly salivary glands, because hybrids were found only in trypanosome populations derived from the salivary glands, not midguts, in analysis of crosses using selectable drug resistance markers [10,11]. However, this approach did not identify the life cycle stage involved, since detection relied on outgrowth of double-drug resistant hybrids and therefore only procyclics and metacyclics (via bloodstream forms) were actually examined. These results also leave open the possibility that mating occurs not in the salivary glands but en route, among the migratory forms (asymmetric dividers, long and short epimastigotes [9,12]) that travel from the proventriculus at the anterior end of the midgut, through the foregut and thence to the salivary glands.

What happens during trypanosome mating remains a mystery; no-one has observed it directly and our current knowledge relies on genotypic comparisons of parents and progeny. Mendelian inheritance of genetic markers in hybrid progeny points to the occurrence of a meiotic division during genetic exchange in *T. brucei *[5,13-18], although a naturally occurring haploid stage has not been observed [19,20]. Most hybrid progeny are diploid like the parental trypanosomes, but triploid hybrids also occur [10,11,13,21-23]. The observation that kinetoplast (mitochondrial) DNA is inherited from both parents in hybrid progeny [24-26] supports the hypothesis that fusion of the parental mitochondria, and hence cells, occurs during genetic exchange. Thus, genetic exchange in *T. brucei *involves both meiosis and fusion, but the order of these events is uncertain [5,27]. The genomes of both *T. brucei *and *T. cruzi *contain meiosis-specific genes [28], but for *T. cruzi*, although fewer crosses have been done, the evidence points to fusion of the parental trypanosomes without meiosis, as hybrids inherited both parental alleles at a number of loci [3].

To find out more about the mechanism of genetic exchange in *T. brucei*, we need to examine intermediate stages in the process. The first step is to pinpoint the developmental stage and the region of the fly where genetic exchange takes place and for this we have developed approaches based on detection of fluorescent hybrids. Initial attempts based on segregation of reporter and repressor genes in hybrid progeny were successful but problematic, as spontaneous mutations of the repressor system leading to reporter gene expression could not be ruled out [29], and thus we could not be certain that all fluorescent trypanosomes were hybrid. A simpler system based on the co-expression of two reporter genes, green and red fluorescent proteins (GFP and RFP), allows hybrid progeny to be detected by dual fluorescence, ie. appear yellow by fluorescence microscopy in contrast to the green or red parental trypanosomes. This system has proved to be successful for the production and easy visualization of hybrids, as briefly reported in [30], and here we present a detailed analysis of four independent crosses.

## Results and Discussion

### Experimental cross between red and green trypanosomes

**Figure 1:**
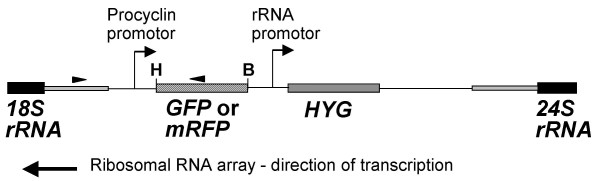
**Diagram of reporter constructs**. Diagram of reporter construct integrated into the non-transcribed spacer of the ribosomal DNA locus of *Trypanosoma brucei*. The *GFP *and *mRFP *genes were exchanged as a *Hin*dIII-*Bam*HI cassette. The boxes represent the genes indicated and the grey boxes represent the rRNA intergenic region, part of which was used for targeting the construct to this location. Thin line represents plasmid construct. The arrowheads show positions of primers used to check integration site. H = *Hin*dIII, B = *Bam*HI.

To visualize the production of hybrids in the fly, parental trypanosome clones were transfected with a gene encoding Green Fluorescent Protein (GFP) or Red Fluorescent Protein (RFP). The correct integration of the *GFP *and *mRFP *constructs into the rRNA locus was confirmed by PCR across border regions (Fig 1). We previously established that the *GFP *reporter gene was expressed throughout the trypanosome developmental cycle in the tsetse fly [29], and confirmed that this was also the case for *mRFP *by experimental fly transmission of the red trypanosome line. A series of crosses was set up by mixing approximately equal numbers of bloodstream forms of the red and green trypanosome lines with the first bloodmeal fed to groups of recently emerged (24–48 hours) tsetse flies. Flies were dissected 3–63 days later and examined for trypanosome infection; a detailed description of the infection results is presented in our companion paper [31], which examined the dynamics of co-infection with the red and green trypanosome clones, and therefore only details relevant to the experimental cross are included here. While almost all midgut infections examined consisted of a mixture of red and green trypanosomes, the composition of the salivary gland trypanosome populations was highly variable, often with disparity between the two glands of the pair (Table 1). We assume that this reflects characteristics of the colonization process, whereby each salivary gland is invaded separately by a small number of migrating trypanosomes which serves as a founder population. Only 22 of 60 flies (36.7%) had a mixture of red and green trypanosomes in one or both salivary glands, a far lower proportion than expected considering the high rate of mixed midgut infections.

**Table 1:** Salivary gland infections

Salivary gland trypanosome infection	No. of flies	No. of flies with yellow trypanosomes
Both glands mixed	13	10
1 mixed, 1 green	5	4
1 mixed, 1 red	3	2
1 mixed, 1 uninfected	1	1
1 green, 1 red	4	0
Both green	11	0
Both red	2	0
1 green, 1 uninfected	11	0
1 red, 1 uninfected	10	0

**Total**	**60**	**17**

**Figure 2:**
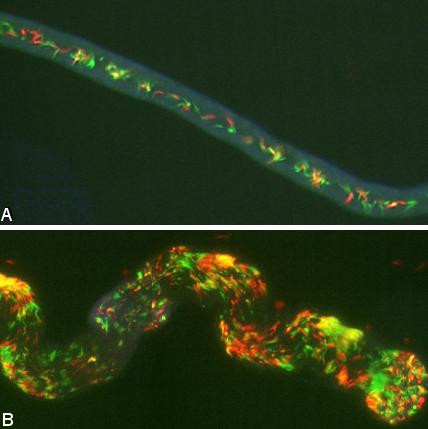
**Yellow hybrids in salivary glands**. Dissected salivary glands with mixed infection of red, green and yellow trypanosomes. A. Salivary duct from 27 day infection. B. Portion of salivary gland showing blind end from 20 day infection. Reproduced from [30] with permission. Trypanosomes are 20–30 μm in length.

**Figure 3:**
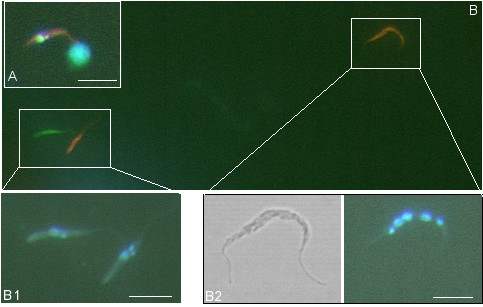
**Yellow fluorescent trypanosomes recovered from saliva or glands**. A. Yellow epimastigote from fly salivary gland dissected at 13 days stained with Hoechst 33342. B. Trypanosomes in a salivary exudate sample obtained from an infected fly at day 14; main picture shows the unfixed trypanosomes by fluorescence microscopy and insets show Hoechst 33342 and brightfield images. B1 are unattached epimastigotes and the yellow epimastigote has two kinetoplasts; B2 appears to be a multinucleate cell. Scale bar = 10 μm.

Yellow hybrid trypanosomes were found either inside or spilling out of the salivary glands of 17 flies, all of which had a mixture of red and green trypanosomes in one or both glands (Table 1; Fig. 2). Yellow trypanosomes were not observed in salivary glands containing only red or only green trypanosomes, even when the other gland of the pair contained a mixed infection of both parents (8/60: 3 flies with 1 mixed + 1 red gland, 5 flies with 1 mixed + 1 green gland), or when both parents were present in the pair of glands, but not each individual gland, ie. one gland with green trypanosomes only and one with red trypanosomes only (4/60) (Table 1; [31]). This demonstrates that mating takes place among trypanosomes that have reached the salivary ducts or glands and not among those able to mix en route in the foregut or mouthparts. The fact that yellow hybrids were found in 17 of the 22 flies in which one or both salivary glands had a mixed infection, suggests that all glands containing a mixture of parental trypanosomes would eventually produce hybrids; in this experiment, mixed glands without yellow trypanosomes had either been dissected at early timepoints (14 – 17 days), or had very low numbers of one of the parents. The earliest timepoint at which yellow trypanosomes were observed in the salivary glands was 13 days after infection; in previous crosses, hybrids have generally appeared at least 28 days after infection [5,7]. These yellow trypanosomes were epimastigotes, as shown by the morphology and close proximity of the kinetoplast to the nucleus (Fig 3). Also, during live imaging, yellow trypanosomes were seen that were clearly attached inside the intact salivary gland (see additional file 1: Movie1), confirming that hybrid formation had occurred by the attached epimastigote stage. On no occasion were yellow trypanosomes observed among midgut trypanosomes: 411 midguts with a mixture of red and green trypanosomes were examined [31]. Nor were yellow trypanosomes found among the foregut migratory forms on their way from the proventriculus to the salivary glands; these developmental stages (proventricular trypomastigotes, asymmetric dividers, short and long unattached epimastigotes) were examined in the salivary exudate from 58 individual flies (see next section).

From all the above evidence, we conclude that mating occurs in situ in the salivary glands and not among migratory forms en route to the salivary glands. The most likely candidate for the sexual stage is the short epimastigote soon after this stage invades the glands, as yellow epimastigotes were observed at the very early timepoint of 13 days.

### Intermediate stages

We searched for intermediate stages at early timepoints during salivary gland invasion and colonization. Individual flies were monitored for production of trypanosomes in samples of salivary exudate (a mixture of saliva and regurgitated foregut contents) from day 8 to 28 after infection by inducing them to probe onto a warm microscope slide [32], and finally examined for trypanosome infection of the salivary glands by dissection 13–28 days after infection, as previously reported [31]. Of 58 flies which extruded trypanosomes in the salivary exudate on one or more occasions, 34 (59%) produced both red and green trypanosomes simultaneously, and one of these flies also produced yellow trypanosomes in a sample obtained on day 14 after infection, comprising one unattached epimastigote with 2 kinetoplasts, presumably in cell division, and a multinucleate trypanosome (Fig 3B). Two multinucleate cells were also found in a mixed gland containing yellow hybrids dissected on day 15 after infection (not shown). However, these were the only cells with unusual morphology noticed and all other trypanosomes examined in salivary exudate or dissected glands corresponded to the expected morphological stages, namely migratory forms (proventricular trypomastigotes, asymmetric dividers, short and long unattached epimastigotes) and salivary gland epimastigotes and metacyclics.

**Figure 4:**
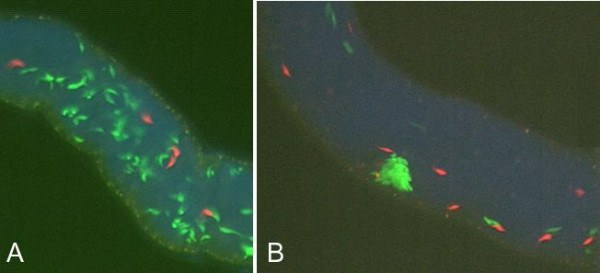
**Early establishment in salivary glands**. Close proximity of red and green trypanosomes in salivary glands at early establishment. Flies dissected at A. 20 days and B. 15 days after infection. Trypanosomes are 20–30 μm in length.

As reported previously [31], only 15 (26%) of the 58 flies that extruded trypanosomes in the salivary exudate were found to have established a salivary gland infection; of these, six contained a mixed infection and five also had yellow trypanosomes; the other fly was dissected at an early stage of salivary gland colonization. In the mixed glands red and green trypanosomes were occasionally seen in close proximity (e.g. Fig 4), but otherwise appeared to be typical epimastigotes. Thus, other than occasional multinucleate trypanosomes, we observed no candidate intermediate stages in genetic exchange. The comparative rarity of putative intermediate stages in flies at early stages of salivary gland colonization contrasts with the ready observation of hybrid trypanosomes in older flies and suggests that trypanosome mating may be a transient and rapid event. Detailed investigation of epimastigotes attached inside the salivary glands at this early stage of establishment was not attempted due to their small number, inaccessibility to reagents and obscuration by the much larger fly epithelial cells.

### Analysis of progeny clones

Trypanosome populations from four flies with mixed salivary gland infections (three flies dissected at 4 weeks, one at 8 weeks) were analysed in detail. Each population should represent the progeny of one independent genetic cross at minimum. Red, green, yellow and/or non-fluorescent clones were recovered from each of the 4 trypanosome populations, giving a total of 63 clones. All clones were checked by PCR for presence of the *mRFP *and/or *GFP *genes, and for correct integration of these genes by PCR analysis of flanking regions (not shown). All yellow clones contained both *mRFP *and *GFP *genes as expected, while the presence of neither gene could be demonstrated in the non-fluorescent clones. The reporter genes were confirmed to be in the ribosomal locus by PCR using a flanking primer paired with a primer in the reporter gene (Fig 1; results not shown).

**Figure 5:**
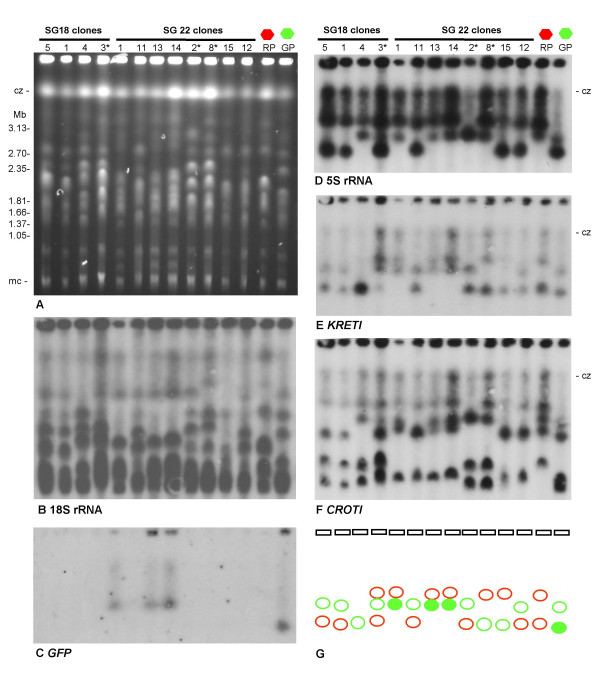
**Comparison of green parental and progeny clones by PFG electrophoresis**. A. Ethidium bromide stained gel comparing the molecular karyotypes of parental and progeny clones, including red parent J10 (RP) and green parent 1738 (GP). The other lanes show the karyotypes of various hybrid progeny clones from 2 different flies; asterisks denote clones with a 4N DNA content; all other hybrid clones and the parents had 2N DNA contents. Size marker: chromosomal DNAs from *Hansenula wingei*. Cz = compression zone, a region of the gel where several large chromosomal bands are trapped. Mc = minichromosomes of approx. 100 kb in size. B – F. Autoradiographs showing results following hybridization with the probes indicated. All blots were washed to 0.1 × SSC at 65°C. G. Diagram indicating the *KRET1 *chromosomal band (filled) that also hybridized with the *GFP *probe in parent 1738 and hybrid progeny, Fly 22 clones 1, 13 and 14. The origins of each chromosomal band have been arbitrarily assigned to either the red parent J10 (red circles) or the green parent 1738 (green circles) according to size; each hybrid clone can be deemed to have inherited one chromosome from each parent, except hybrid clone Fly 18–3, which has an extra chromosomal band that may have originated from either parent. This clone also has extra chromosomal bands in hybridizations for chromosomes IV (*CROTI*) and VIII (5S rRNA; *CROTI*).

**Figure 6:**
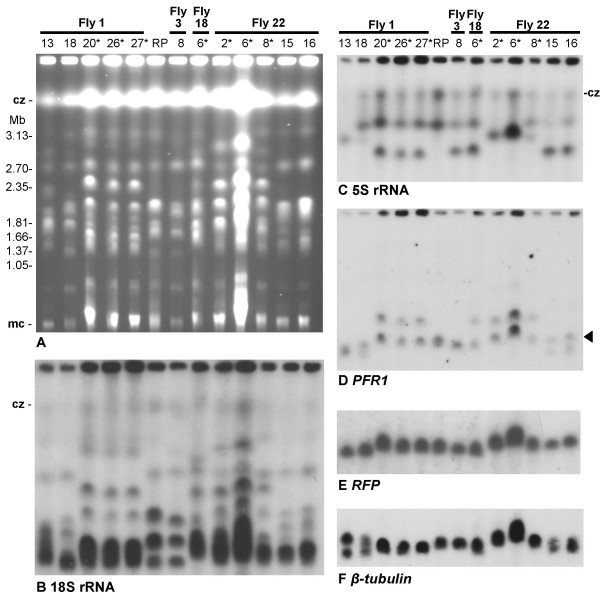
**Comparison of red parental and progeny clones by PFG electrophoresis**. A. Ethidium bromide stained gel comparing the molecular karyotypes of red fluorescent clones, including red parent J10 (RP). The other lanes show the karyotypes of various hybrid progeny clones from 4 different flies. Size marker: chromosomal DNAs from *Hansenula wingei*. Cz = compression zone, a region of the gel where several large chromosomal bands are trapped. Mc = minichromosomes of approx. 100 kb in size. B – F. Autoradiographs showing results following hybridization with the probes indicated. All blots were washed to 0.1 × SSC at 65°C. The arrow (panel D) indicates the *PFR1 *chromosomal band that also hybridizes with the *RFP *probe (panel E). The asterisked clones had 4N DNA contents; all other clones were diploid like the parents except for Fly 1 clone 18, with DNA content 3N. These hybridization results give no hint that any of these clones, whatever its DNA content, has extra chromosome bands.

Molecular karyotypes of parental and progeny clones were compared by pulsed field gel electrophoresis (PFG). All progeny clones showed a non-parental karyotype, whatever their colour (e.g. Figs 5A and 6A), as a result of reassortment and recombination of parental chromosomes. It was evident that some progeny clones from the same fly had identical karyotypes (e.g. Fly 18 clones 1 and 5, and Fly 22 clones 1, 13 and 14, Fig 5A; Fly 1 clones 20, 26 and 27, Fly 22 clones 2 and 6, and clones 15 and 16, Fig 6A); these observations were borne out by hybridization with a probe for 18S rDNA, which is carried on several different chromosomes (arrays on chromosomes II, III and VII [33], e.g. Fig 5B and 6B). *GFP *and *mRFP *genes were localized to different chromosomes carrying rDNA arrays by hybridization, with *mRFP *on chromosome III and *GFP *on chromosome VII (Figs 5 and 6). Individual chromosomes were identified by localization of genes for *β-tubulin *(chromosome I), *PFR1 *(chromosome III), *CROT1 *(chromosomes IV and VIII), *KRET1 *(chromosome VII), 5S rRNA (chromosome VIII). The presence of the *GFP *or *mRFP *gene served to distinguish the two homologues of the pair, and it was obvious that *GFP *had switched from its original position on the smaller chromosome VII homologue (VIIa) in parent 1738 to the larger homologue (VIIb) in some hybrid clones (e.g. Fly 22 clones 1, 13 and 14, Fig 5C, 5G); of 5 green or yellow hybrid genotypes examined, 1 had *GFP *on VIIa and 4 had *GFP *on VIIb. There was also some evidence of size variation in the chromosome III homologue carrying the *mRFP *gene (e.g. Fig 6E), but the small differences in size between chromosome III homologues, coupled with run perturbations of individual gels, made this difficult to establish unequivocally. Extra chromosomal bands are clearly visible in the hybridization results for Fly 18 clone 3 (Fig 5 panels D, E and F); this clone was found to have a 4N DNA content (see below).

**Figure 7:**
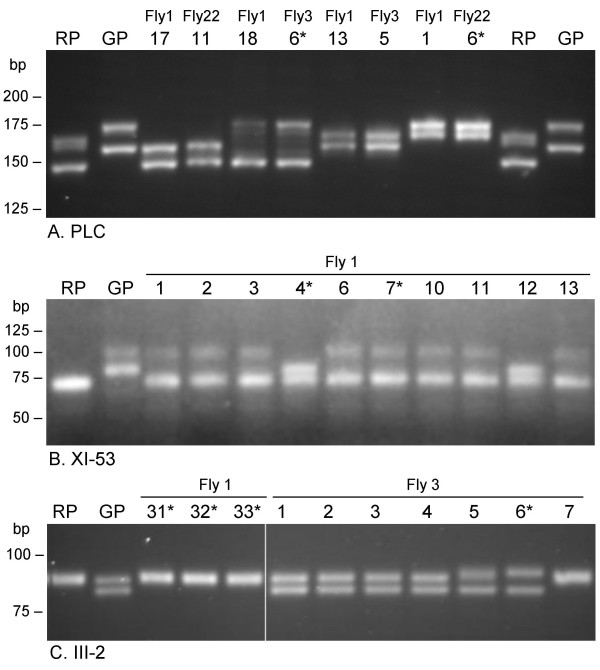
**PCR amplification of microsatellite alleles**. Results of PCR amplification of 3 microsatellite loci. RP = red parent J10; GP = green parent 1738; other lanes show alleles amplified from individual hybrid progeny clones from 3 flies. Both parental trypanosomes are heterozygotic at the II-PLC and III-2 loci, but only GP is heterozygotic at the XI-53 locus; for II-PLC the larger allele of RP appears as 2 closely spaced bands, but is inherited as a single band in progeny clones. The parental origin of each allele can be unequivocally identified by size for II-PLC, but for XI-53 RP is homozygous and for III-2, alleles a, b and d are closely similar in size and required long gel runs and application of samples in adjacent lanes to be distinguished. Each progeny clone has 2 strongly amplified bands and appears to have inherited one allele from each parent. Asterisked clones had 4N DNA contents; all other clones and parents were diploid, except for Fly 1 clone 18, which had 3N DNA content.

Microsatellite analysis confirmed that all progeny clones were hybrid, since each had inherited one allele from each parent at the II-PLC, III-2 or XI-53 loci (Fig 7); these microsatellite markers are on chromosomes II, III and XI respectively. In all but one of the hybrid progeny clones, the inheritance of these markers was consistent with Mendelian reassortment of unlinked genes in diploid F1 progeny, ie. each clone had one allele from each parent at each locus. The exception was a red clone, Fly 3 clone 8, for which the microsatellite profile was identical to that of parent J10 for 2 loci (genotype XIX, Table 2). The karyotype of this clone was similar but not identical to that of J10 (Fig 6A) and chromosomal differences were evident from hybridization with rDNA probes (Figs 6B, 6C). This clone also had kinetoplast DNA (kDNA) from the 1738 parent (see below) and therefore without doubt is a genetic hybrid. The simplest explanation for the anomalous microsatellite genotype of this clone is that it resulted from more than a single round of mating.

**Table 2:** Genotypes and phenotypes of hybrid clones and parents

**Genotype (no. of clones)**	**Fluorescence**	**Microsatellite alleles**			**Maxicircle type (no. of clones)**	**DNA content**
				
		**II-PLC**	**XI-53**	**III-2**		
J10 mRFP	Red	ab	aa	ab	J10	2N
1738 GFP	Green	cd	bc	cd	1738	2N
**SG1**						
I (3)	Green	bd	ac	ac	J10	2N
II (1)	Green	bd	ac	bc	1738	2N
III (1)	Green	bc	ac	bc	1738	2N
IV (5)	None	bd	ac	ac	1738	2N
V (1)	None	bd	ac	ac	mix	2N
VI (1)	None	bd	ab	ac	1738	2N
VII (1)	None	bd	ac	ad	1738	4N + 2N
VIII (1)	None	ac	ab	ad	J10	2N
IX (3)	None	bd	ac	ac	1738	2N
X (2)	None	bd	ab	bc	1738	2N
XI (1)	Red	bc	ac	ac	1738	2N
XII (1)	Red	ad	ac	bd	1738	3N
XIII (3)	Red	ad	ac	bc	1738	4N + 2N
XIV (1)	Yellow	bd	ab	ad	J10	4N + 2N
XV (8)	Yellow	ad	ac	bd	1738	4N + 2N
**SG3**						
XVI (4)	None	bc	ac	ac	1738 (3), mix (1)	2N
XVII (1)	None	ad	ab	bc	1738	4N + 2N
XVIII (1)	Red	bc	ab	bc	J10	2N
XIX (1)	Red	ab	aa	ad	1738	2N
XX (1)	Yellow	bc	ab	bd	mix	2N
**SG18**						
XXI (3)	None	bc	ab	bc	J10 (2), 1738 (1)	2N
XXII (1)	None	ac	ab	bd	1738	4N
XXIII (1)	None	bc	ab	ac	J10	2N
XXIV (1)	Red	ac	ac	bc	1738	4N
**SG22**						
XXV (9)	Green	bc	ac	ad	J10 (6), mix (3)	2N
XXVI (2)	None	ac	ab	ad	J10	2N
XXVII (2)	Red	bd	ac	bd	J10	4N + 2N
XXVIII (1)	Red	ac	ab	bc	J10	4N + 2N
XXIX (2)	Red	bc	ac	bc	mix	2N

The 63 progeny clones resolved into 29 different genotypes by combining the karyotype and microsatellite results (Table 2). Analysis of the allele frequencies for the 29 genotypes revealed no deviation from Hardy-Weinberg expectations (data not shown). As the III-2 microsatellite locus is on the same chromosome as the *mRFP *gene, one III-2 allele should co-segregate with the reporter gene. Allele *a *occurs in 3 genotypes with *mRFP *and 11 without, while allele *b *occurs in 9 genotypes with *mRFP *and 6 without (Table 2). This suggests that there has been frequent recombination between the III-2 and rRNA loci on chromosome III. On the map of chromosome III [34] these 2 loci are separated by approximately 600 kb.

### Kinetoplast DNA inheritance

**Figure 8:**
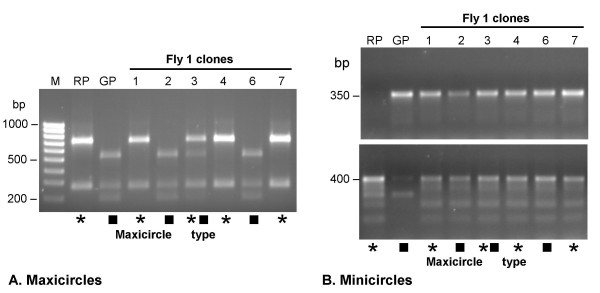
**Analysis of kinetoplast DNA inheritance**. A. Maxicircle fragments resulting from *Hin*fI digestion of PCR amplified *COXI *gene. * J10 (RP) maxicircle type; ■ 1738 (GP) maxicircle type. Inheritance is uniparental except for Fly 1 clone 3, which has maxicircles of both parental types; this result was confirmed in a second reaction. B. PCR products resulting from amplification of genomic DNA using two different sets of minicircle-specific primers. All hybrid progeny clones have the 1738 (GP) minicircle (top) and the J10 (RP) minicircle (bottom) notwithstanding the type of maxicircles inherited from the parents (indicated by the * and ■ symbols as above).

Inheritance of kDNA mini- and maxicircles was examined by PCR analysis and restriction digestion (Fig 8). The parental trypanosomes differed in a polymorphic *Hin*fI restriction site in the maxicircle gene for cytochrome oxidase (*COXI*), allowing the maxicircle type of individual progeny clones to be determined. While most clones had only a single parental maxicircle type, a few had both parental maxicircles present (Fig 8A). We have not previously detected mixed maxicircle networks in hybrid clones, although this was reported by others [25]; this probably results from the greater sensitivity of PCR as a detection method compared to direct analysis of purified kDNA as done previously [24,26].

Individual minicircles from the parental trypanosomes were cloned and sequenced in order to design primers for PCR detection of specific minicircles. Minicircles from both parents could be seen in all progeny clones (Fig 8B), showing that minicircle networks are hybrid whatever the maxicircle type. The presence of kDNA elements attributable to both parents in progeny clones is evidence that mitochondria and therefore whole cells fuse during mating. Bi- and uniparental maxicircle inheritance fits with the hypothesis that the kDNA networks fuse during mating, generating an initial network containing both maxicircle types, which subsequently resolves to a single maxicircle type by progressive vegetative divisions [24,25]; the minicircle network remains mixed due to the large number and variety of minicircles present. This idea is supported by the observation that some clones with an identical genotype by karyotype and microsatellite analyses (above) had maxicircles of different parental type, e.g. Fly 18 clones 1, 5 and 7. A recent result showing that maxicircles are held in a bridge structure between separating kDNA networks during division [35], points to the possibility that a more sophisticated mechanism may operate during resolution of hybrid networks.

There did not appear to be any bias for inheritance of one parental maxicircle type: 11 hybrid genotypes inherited J10, 18 inherited 1738, and 5 inherited both parental types (total > 29, as some hybrid genotypes differed in maxicircle inheritance, as mentioned above). It is noteworthy that the hybrid clone that was closely similar to the J10 parent, Fly 3 clone 8, had maxicircles of the other parental type and a hybrid minicircle network.

### DNA contents

DNA contents of progeny and parental clones were measured by flow cytometry of fixed and permeabilized procyclic cells stained with propidium iodide. The 488 nm laser of the flow cytometer excites propidium iodide but not mRFP, and we confirmed that unstained parental red or green fluorescent trypanosomes gave identical background levels of fluorescence to wildtype. On an initial flow cytometer run, the DNA contents of both parents and a representative clone from each of the 29 hybrid genotypes was measured; on subsequent runs, measurements were repeated on regrown samples of the parents and selected hybrid clones, either the clone used on the first run or another clone of the same genotype, to check that results were reproducible.

**Figure 9:**
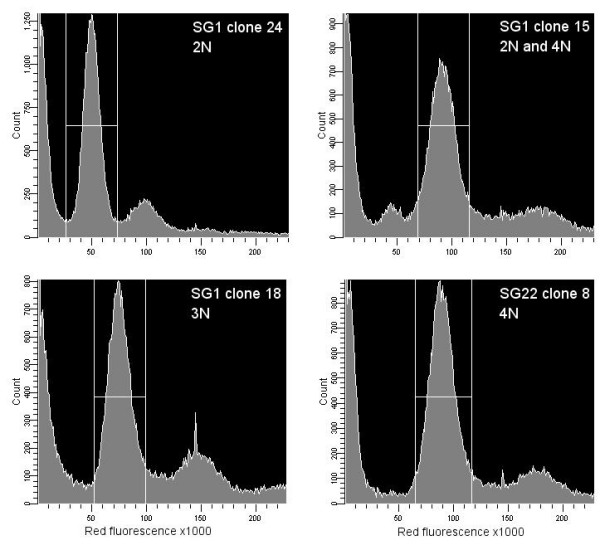
**Flow cytometry histograms**. Flow cytometry histograms for 4 hybrid trypanosome clones as indicated. Red fluorescence in arbitrary units (x axis) against cell count (y axis). The G1 peak for each histogram is gated; an additional smaller G1 peak is present in SG1 clone 15.

**Figure 10:**
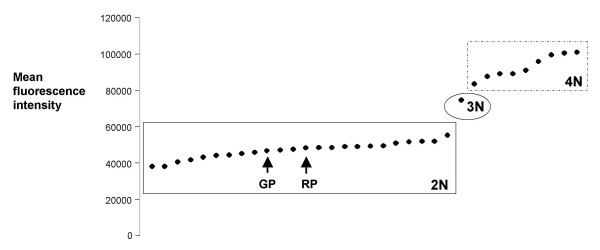
**Analysis of DNA contents by flow cytometry**. Values for mean red fluorescence intensity of the G1 peak of each histogram, representing the DNA content of the trypanosome clone, are plotted in ascending order. The values for the diploid J10 (RP) and 1738 (GP) parents are indicated. Values for hybrid clones within the solid box are also consistent with diploidy (mean 47042, s.d. 4364). Values for hybrid clones within the hatched box correspond with 4N (mean 94083, s.d. 13091); the remaining clone (circled, Fly 1 clone 18) had a value of 74611, corresponding to a 3N DNA content.

Most progeny clones had approximately the same DNA content as the parents, consistent with diploidy (Fig 9). However, many polyploid clones were also identified, where the G1 peak was consistent with 3N or 4N (Fig 10). Although 3N hybrids have been reported in several of our previous crosses, e.g. [22], 4N progeny have not been observed except possibly in the first experimental cross reported, where it was thought that fusion of diploids produced an unstable tetraploid [13]. Of the 29 genotypes of hybrid progeny, 19 had DNA contents consistent with 2N (4 green, 4 red, 1 yellow, 10 no fluorescence), 1 with 3N (red) and 9 with 4N (4 red, 2 yellow, 3 no fluorescence); polyploid genotypes (consistent with 3N or 4N) were found among hybrid progeny from all 4 flies examined and thus had been generated independently in a minimum of 4 crosses (Table 2).

The 4N G1 peak was consistently found in repeated runs of the same clone or clones of the same genotype, but histograms sometimes also had a smaller peak equivalent to a 2N G1 peak (e.g. SG1 clone 15, Fig 9), interpreted as a mixture of diploid and tetraploid cells. This may indicate reduction of the tetraploid cell to diploid as reported earlier for *T. brucei *[13] and described for the yeast *Candida albicans *[36]. This requires further investigation of longterm cultures of polyploid clones.

Considering the results overall, the majority of hybrid genotypes fit with a conventional meiotic division of a diploid cell, with Mendelian patterns of inheritance demonstrated by microsatellite alleles and homologous chromosomes. However, an uncomfortably large number of hybrid genotypes (10 of 29) do not fit this model, because they have DNA contents consistent with polyploidy. Both sorts of hybrid (diploid and polyploid) were found in all 4 independent crosses examined. Just like the diploid clones, the polyploid hybrid clones uniformly showed 2 amplified bands for the II-PLC and III-2 microsatellite loci, for which assignment of parental alleles was unequivocal (J10 is homozygotic for the XI-53 locus). Simple fusion of the 2 parental genomes, as recorded for *T. cruzi *[3], would have resulted in hybrid progeny with 4 alleles at each microsatellite locus and evidence of extra chromosomes in PFG analysis. However, extra chromosomal bands were demonstrated in only one 4N genotype, Fly 18 clone 3, which had a minimum of 3 copies of chromosomes IV, VII and VIII (Fig 5 panels D, E and F). Of course, the presence of extra chromosomal bands will sometimes be hidden by co-migration of chromosomal bands on the PFG gels, but this has occurred rather too often here to be a satisfactory explanation. A total of 5 different chromosomes (I, III, IV, VII, VIII) were screened in the PFG analysis with sufficient size variation between parental homologues of chromosomes III, IV, VII and VIII to facilitate detection of aneuploidy. Taking the microsatellite and PFG results together, the inescapable conclusion is that individual parental homologues have been duplicated in these clones.

Can the polyploid genotypes have been generated through errors of a conventional sexual cycle or are they the result of a separate parasexual process? We have previously argued that 3N hybrids result from fusion of diploid and haploid nuclei and thus support, rather than conflict with, meiosis as part of the process of genetic exchange in *T. brucei *[10]. One of the 4N genotypes analysed here (Fly 18 clone 3) also fits into this scheme as it has the extra chromosomal bands expected from fusion of randomly selected diploid or haploid cells/nuclei, but the majority of 4N genotypes look more like the products of genome endoreplication. In other organisms, polyploidy quite commonly arises in hybrid taxa and such taxa produce unreduced gametes at high frequency, which is thought to be the result of problems in pairing homologous chromosomes that have diverged in sequence in the separate taxa before hybridization [37]. This suggests another explanation for the large proportion of *T. brucei *polyploids observed here, if *T. brucei *homologues from different strains are sufficiently divergent to cause failure of meiotic pairing. This might happen on the first round of mating if parental genomes fuse before meiosis, or on subsequent rounds when F1 hybrids attempt to undergo meiosis. Although there is no data on the extent of chromosome divergence between the trypanosome strains used here, divergence in the subtelomeric arrays of VSG genes [33], both in size and sequence, would surely be expected, even on homologues from a single strain. This would also account for the variability in the frequency of polyploids observed in different laboratory crosses, as a variety of trypanosome strains have been used. Thus the formation of all the diploid and polyploid hybrids described here can be explained by a model involving meiosis, and this remains the simplest hypothesis.

Since the genotypes of all the diploid clones from these crosses (except Fly 3 clone 8) conform to expectations for the F1 generation, we have assumed that only a single round of mating has taken place, but there is no a priori reason to believe this; moreover, the genotype of Fly 3 clone 8 is more readily explained as the product of an F1 rather than parental cross. The high frequency of polyploid hybrids might also arise from mating among F1 hybrids, if this leads to problems in meiotic pairing of homologous chromosomes as discussed above. On the other hand, if only the short epimastigotes newly arrived in the salivary glands can mate, then this would favour an initial burst of mating followed by sporadic events if further migratory trypanosomes reach the salivary glands.

Both 2N and 4N clones were successfully transmitted through tsetse, demonstrating that polyploidy does not compromise completion of the full developmental cycle. The morphology of 4N cells was comparable with that of 2N cells, although the nucleus appeared to be slightly larger in the 4N cells (not shown). However, it is doubtful whether such polyploid trypanosomes would persist if produced in nature, unless polyploidy conferred sufficient advantages to outweigh the cost of replicating twice as much DNA, for example by doubling the available repertoire of *VSG *genes. This could also be achieved by expansion of individual chromosomal *VSG *arrays though, and the high proportion of pseudogenes in these arrays already points to combinatorial diversity as a key mechanism to increase the antigen repertoire [33]. While the DNA contents of *T. cruzi *strains vary markedly in nature [38,39], much lower levels of variation have been reported in *T. b. brucei *[40]. On the other hand, comparative analysis of trypanosome genome sequences indicates that a major duplication event involving chromosomes IV and VIII occurred at some stage in the evolutionary history of *T. brucei *that evidently conferred sufficient benefit to persist [41].

## Conclusion

In this experimental *Trypanosoma brucei *cross, genetic exchange readily occurred when the two different strains were together in the same salivary gland, but did not occur in other infected organs despite very high densities of parental trypanosomes. Trypanosomes therefore need to reach the salivary ducts or glands before they can mate. This, together with the fact that the first hybrid cells observed were epimastigotes, indicates that the life cycle stage that mates is the unattached epimastigote. Relatively few short epimastigotes constitute the founder population that invades and colonizes each salivary gland [31] and the early occurrence (13 days) of hybrid trypanosomes suggests that these trypanosomes mate and then rapidly populate the salivary glands, leading to the predominance of hybrid over parental genotypes observed here. It seems that the main barrier to genetic exchange is not reluctance of trypanosomes to mate, but rather the low probability of both parental trypanosomes reaching the same salivary gland.

Results from the genetic analysis of hybrid clones were not entirely consistent with a simple model based on meiosis of a diploid cell and Mendelian inheritance of markers, because a high proportion of hybrids were polyploid. The genotypes of these clones were not consistent with simple parental fusion as described in *T. cruzi *[3], and most probably result from a high frequency of formation of unreduced gametes during meiosis. All results from the *T. brucei *crosses described here can therefore be fitted into a model involving meiosis.

## Methods

### Transfection

The reporter construct pHD67E containing the *GFP *gene was described previously [29]. The *GFP *gene was replaced by a *Hin*dIII-*Bam*HI cassette containing the *mRFP *gene from a construct kindly supplied by Roger Tsien [42]. Plasmid DNA was purified from bacterial cultures using commercial midiprep kits and trypanosomes were transfected as described previously [29]. Mid-log phase procyclic trypanosomes of isolate J10 (*T. b. brucei *MCRO/ZM/73/J10 CLONE 1; [43]) or 1738 (*T. b. brucei *MOVS/KE/70/1738; [44]), grown in Cunningham's medium [45] supplemented with 10% v/v heat-inactivated foetal calf serum, 5 μg/ml hemin and 10 μg/ml gentamycin (complete medium = CM) at 27°C, were transfected with reporter constructs by electroporation using two pulses of 1.5 kV, 25 μF. Note that the full names of J10 and 1738 are given correctly here. Transfectants were selected 24 hours post-electroporation by the addition of Hygromycin B, 50 μg/ml. The population was checked for fluorescence by microscopy of living cells. Clones were obtained by two rounds of limiting dilution of procyclics in CM in 96 well plates incubated at 27°C in 5% CO_2_.

### Tsetse transmission and experimental cross

Trypanosomes were transmitted through male tsetse flies (*Glossina morsitans morsitans*) as described previously [17,31], supplementing the infective bloodmeal with 60 mM N-acetylglucosamine to increase infection rates [46]. Infected flies were maintained on membrane-fed sterile horse blood supplemented with 2.5% w/v bovine serum albumen (Sigma A4503) [47] and 1 mM dATP [48]. Flies were dissected up to 9 weeks following the infective feed. Metacyclics from infected salivary glands were inoculated into mice; bloodstream forms were subsequently transformed back to procyclics to facilitate cloning and preparation of samples by incubation in CM at 27°C.

### Microscopy

Living trypanosomes were viewed as wet mounts in CM, blood or phosphate buffered saline (PBS). Whole tsetse midguts or salivary glands were dissected into a drop of PBS and viewed as wet mounts. Dried saliva samples were obtained by allowing flies to probe onto a warm microscope slide before feeding [31,32]. Cells were fixed in 2% w/v paraformaldehyde at room temperature for 20 minutes and stained with a 1/100 dilution of Hoechst 33342 for 30 minutes to visualize the nucleus and kinetoplast, if required. A DMRB microscope (Leica) equipped with a Colour Coolview camera (Photonic Science) was used for fluorescence and standard microscopy, with ImagePro Plus software (Media Cybernetics).

### Genotype analysis

Genomic DNA samples from *T. brucei *were prepared from approximately 5 × 10^7 ^washed procyclics using a spin column DNA purification kit (Qiagen). Samples for pulsed field gel electrophoresis (PFG) were prepared by lysing and deproteinising trypanosomes in situ in agarose blocks [49]. PCR was performed by standard methods using the following primers: EGFP-R 5' TCAGCTTGCCGTAGGTGG, RFP-R 5' CTCGATCTCGAACTCGTG, SSUsp-G 5' CATGCAACAGTACACTTCAC. Microsatellite alleles were amplified by PCR as described [18] using primers PLC-G 5' CAACGACGTTGGAAGAGTGTGAAC, PLC-H3 5' CCACTGACCTTTCATTTGATCGCTTTC, III-2A 5' GGTGGAATGGAAGATCAGTT, III-2B 5' GTTGGAATTGTTGTTGCTGT, XI-53A 5' CGTGTGTCTTGTATATCTTCT, XI-53B 5' TGAATAAACAAAACATGAAACGAC. These 3 loci were selected on the basis of an initial screen of the parental trypanosomes for allelic variation. Products were resolved by electrophoresis in 1 × TAE buffer through 3–5% Metaphor agarose (Cambrex) gels. Chromosomes were separated using a Biorad CHEF-DR III with a 2 phase program (Block 1: switch time 1800 s, voltage 2 V/cm, angle 106°, 15 hours; Block 2: switch time 300–900 s, voltage 3 V/cm, angle 106°, 50 hours) using 0.5 × TBE buffer and 0.9% agarose gels. Gels were stained overnight by submersion in electrophoresis buffer containing ethidium bromide (2 μg/ml). Blotting and hybridization were by standard methods [50,51] using the following PCR-amplified, P^32^-labelled DNA fragments as hybridization probes: *GFP *and *mRFP *genes from the plasmid constructs used for transfection; *β-tubulin *from cDNA plasmid clone [52]; 18S rRNA, *PFR1*, *CROT1*, *KRET1*, 5S rRNA genes from *T. brucei *genomic DNA. Kinetoplast DNA minicircles were amplified using primers designed to individual sequenced minicircles from parental trypanosome J10: J10F-1 5' GTGCAATGCCTCGTAACTAT, J10F-2 5' CCACCCAGAAAGCCTTAT; J10G-1 5' AGCAGTGATTGTTACTTGGG, J10G-2 5' TTTCCTCCTCTACGCACA. Kinetoplast DNA maxicircles were amplified using primers designed to a 982 bp polymorphic region of the cytochrome oxidase subunit I (COI) gene [44] (Accession no. of *T. b. brucei *strain 427 maxicircle = M94286): Max1 5' CCCTACAACAGCACCAAGT, Max2 5' TTCACATGGGTTGATTATGG.

### Measurement of DNA content

Procyclic trypanosomes were harvested from log phase culture, washed 3 times in ice cold Hank's balanced salt solution (HBSS) without Ca^2+ ^or Mg^2+ ^with 0.5 mM EDTA, fixed with 95% ethanol (final concentration 70% ethanol) and stored at 4°C. Fixed cells were pelleted by centrifugation and resuspended in PBS containing 50 μg ml^-1 ^propidium iodide and 40 μg ml^-1 ^RNAse. After incubation at ambient temperature for 30 minutes, cells were recovered by centrifugation and resuspended in PBS. Red fluorescence was measured by flow cytometry using a Beckman FACS DIVA; results were confirmed on a further sample grown from cryopreserved trypanosomes.

## Competing interests

The author(s) declare that they have no competing interests.

## Authors' contributions

WG, LP and MB designed the study. WG transfected the trypanosomes and genotyped the clones with help from KW; LP and VF carried out the tsetse transmission experiments and imaging; WG and MB measured DNA contents; WG and LP drafted the manuscript. All authors read and approved the final manuscript.

## Supplementary Material

Additional file 1 **Movie1. Yellow hybrid trypanosomes in a tsetse salivary gland**. Video clip shows portion of the narrow end of a salivary gland containing yellow, red and green trypanosomes, some of which are attached to the epithelium and are therefore epimastigotes. Click here for file
